# Prehospital Detection of Acute Pulmonary Embolism and Its Association with Clinical Outcomes: A Retrospective Cohort Study

**DOI:** 10.3390/jcm15218426

**Published:** 2026-10-29

**Authors:** Daniel Strohleit, Dominik Brilo, Johanna Treffts, Aydan Ewers, Assem Aweimer, Moritz Seiffert

**Affiliations:** Department of Cardiology and Angiology, BG University Hospital Bergmannsheil Bochum, Ruhr University Bochum, 44789 Bochum, Germany; daniel.strohleit@bergmannsheil.de (D.S.);

**Keywords:** pulmonary embolism, emergency medical services, diagnostic sensitivity

## Abstract

**Background:** Pulmonary embolism (PE) is a potentially life-threatening condition requiring timely diagnosis and treatment. However, data on prehospital correct PE detection by emergency medical services (EMS) are scarce. **Methods:** We conducted a retrospective single-center cohort study at the BG University Hospital Bergmannsheil Bochum, Germany, including all patients with in-hospital confirmed PE (ICD-10: I26.0/I26.9) and admitted by EMS between January 2019 and December 2024. Prehospital PE detection was defined as documentation of PE as the working diagnosis in the EMS protocol. Time to definitive diagnosis was calculated from EMS alarm to computed tomography pulmonary angiography (CTPA) acquisition. Multivariable Cox regression and logistic and linear regression models were used to identify predictors of time to diagnosis and clinical outcomes. **Main Results:** Of 171 patients included, prehospital PE was correctly suspected in only nine cases (5.3%, 95% CI 2.8–9.7%). Time to definitive diagnosis was significantly shorter in patients with correct prehospital PE recognition than in those without (median 2.1 vs. 4.0 h, log-rank *p* < 0.001). In an exploratory analysis, detection occurred only in physician-staffed EMS encounters (10.8% vs. 0%, *p* = 0.003). Prehospital intravenous heparin, administered predominantly for suspected myocardial infarction, was also associated with a higher rate of CTPA acquisition, 2.42 (95% CI 1.40–4.19), likely reflecting confounding by indication. Overall, 56 patients (33.1%) were not diagnosed within 6 h of emergency department presentation; 38 (22.5%) were diagnosed only after more than 24 h. **Conclusions:** Correct detection of PE by EMS is critically low, yet correct detection is associated with earlier imaging. These findings highlight the urgent need for EMS-specific diagnostic strategies and prospective trials to improve prehospital PE detection.

## 1. Introduction

Pulmonary embolism (PE) remains a major contributor to the global disease burden [1,2] despite declining PE-related mortality with geographical heterogeneity [3]. PE-related in-hospital mortality rates range from 6.5% to 13.3% [4,5], especially in elderly patients [6]. According to current guidelines [7], risk stratification of PE into low-risk, intermediate-low-risk, intermediate-high-risk and high-risk is essential. A key component of risk stratification is the pulmonary embolism severity index (PESI) [8,9]. Mortality rises progressively as PESI score increases [10]. Observational data reported 3.4% of patients with PE to be high-risk, 63.6% to be intermediate risk and 33% to be low-risk PE [11,12]. Therapeutic and diagnostic advances over recent years have substantially influenced pulmonary embolism treatment strategies and outcomes, most notably the introduction of direct oral anticoagulants, which are associated with lower bleeding rates [13], and interventional thrombectomy or catheter-directed thrombolysis, which have been shown to reduce hemodynamic deterioration in patients with intermediate-high-risk pulmonary embolism [14]. Despite these advances, a timely diagnosis remains crucial. Evaluation for PE requires an integrated workflow combining clinical history, physical examination, laboratory testing and imaging [15]. Pulmonary embolism has been described as a clinical “chameleon”, given the highly nonspecific and variable nature of its presenting symptoms. Frequent chief complaints include dyspnea and pleuritic chest pain [15], however, syncope or hemoptysis may equally be linked to acute PE [16]. A large retrospective analysis of more than 1.5 million patients found that PE was diagnosed in less than 1% of patients with syncope [17].

Hence, clinicians face a dilemma between the risk of missing a potentially fatal PE and the harms associated with unnecessary diagnostic imaging, such as radiation exposure and contrast-induced complications from CT pulmonary angiography. Most diagnostic strategies have consequently been developed with the primary aim of safely ruling out PE [18]. To improve diagnostic accuracy and standardize clinical decision-making, several PE probability scores have been validated, including the Wells score [19], the revised Geneva score [20], and the Pulmonary Embolism Rule-Out Criteria (PERC) [21]. In a large meta-analysis, clinical decision-making (gestalt) by emergency department physicians using the Wells score alone was insufficient to safely exclude PE and only achieved adequate safety when combined with a sensitive D-dimer cutoff [22]. A recent IPD meta-analysis showed that a positive gestalt was associated with a 3-fold higher risk of PE [23].

Echocardiography plays a distinct role in the diagnostic pathway of acute PE. As a stand-alone diagnostic test, transthoracic echocardiography (TTE) shows consistently high specificity but modest sensitivity for signs of right ventricular strain, with pooled estimates of 53% for sensitivity (95% CI, 45–61%) and 83% for specificity (95% CI, 74–90%) [24]. Echocardiography therefore functions primarily as a “rule-in” rather than a “rule-out” test and cannot be used to exclude PE. Consequently, echocardiography is more valuable for risk stratification than for primary diagnosis.

Most diagnostic approaches aim for the work-up of patients within the emergency department (ED), including echocardiography and age-dependent D-Dimer cutoff values [25]. Regardless of standardized workflows, PE is mis- and underdiagnosed in approximately 27.5% of ED presentations and in roughly half of inpatient cases [26].

To facilitate early diagnosis and treatment of acute PE, successful detection of PE in the prehospital setting may be valuable. Specialized pulmonary embolism response teams (PERT) may be one strategy to improve PE therapy [27]. Whether accurate and early detection of PE by EMS enhances timely treatment or helps allocate patients to appropriate hospitals, potentially improving outcomes, remains unknown. Current guidelines [7,12,15] do not provide EMS-specific recommendations to improve the early detection of acute PE. Hence, we assessed the diagnostic sensitivity for accurate pulmonary embolism detection in the prehospital setting and evaluated potential associations with clinical outcomes.

## 2. Materials and Methods

### 2.1. Study Design

This retrospective, single-center cohort study was approved by the Ethics Committee of the Ärztekammer Westfalen-Lippe and the Faculty of Medicine of Ruhr University Bochum (approval number: 2025-297-f-S). The study was conducted in accordance with the Declaration of Helsinki (last revision 2024) and in accordance with the guidelines of good clinical practice. Due to its strict retrospective design, individual informed consent was waived. A subgroup analysis of a smaller cohort (n = 96) derived from this study, focusing on sex differences and restricted to patients with central PE, has previously been presented in abstract form at a scientific meeting [28].

### 2.2. Study Population

**Figure 1:**
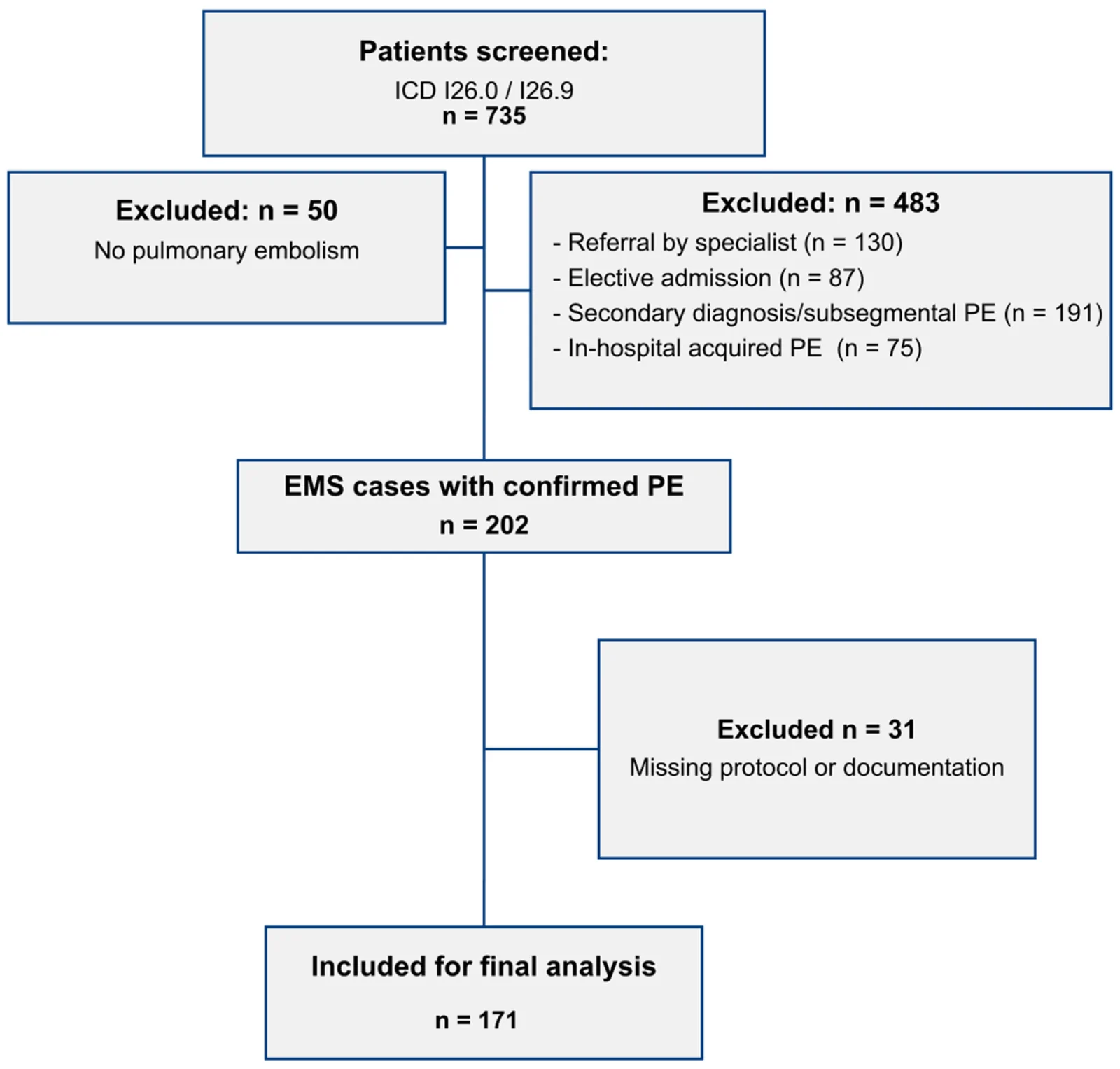
Study flow diagram. Patients were screened from the hospital electronic medical records system using ICD-10 codes I26.0 and I26.9. Numbers in parentheses indicate reasons for exclusion at each step. EMS, emergency medical services; ICD, International Classification of Diseases; PE, pulmonary embolism.

All patients aged ≥18 years admitted to BG University Hospital Bergmannsheil Bochum between January 2019 and December 2024 with a diagnosis of pulmonary embolism (ICD-10: I26.0/I26.9) were identified through the hospital’s electronic medical records system and screened for eligibility. Patients were included if (1) pulmonary embolism was confirmed via computed tomography pulmonary angiography (CTPA) with central or at least multisegmental extent, (2) they were presented by emergency medical services (EMS), and (3) sufficient EMS documentation, including the variable required for the primary endpoint, was available. Patients were excluded if they had already been referred by a specialist with a suspected diagnosis of pulmonary embolism, were transferred from another hospital, acquired pulmonary embolism during in-hospital treatment had missing EMS protocols, or if pulmonary embolism was not the primary reason for hospital admission (Figure 1). In cases with markedly delayed diagnosis, eligibility and attribution of PE to the index EMS encounter were assessed by two physician investigators (D.S. and A.E.) independently, based on the full medical record. In these cases, PE was attributed to the index encounter only if symptoms consistent with PE were documented at EMS contact and no alternative diagnosis explaining the presentation was identified and both reviewers agreed on this attribution. Disagreements were resolved by consensus with a third reviewer (M.S.); cases with persisting uncertainty were excluded.

### 2.3. Data Source and Collection

Data were retrospectively extracted from multiple sources within the hospital information system, including prehospital emergency medical services (EMS) protocols, emergency department documentation, inpatient medical records, and radiology reports (computed tomography pulmonary angiography). All data were manually abstracted by a single trained investigator and entered into a structured GCP-conforming electronic database using REDCap (Research Electronic Data Capture) electronic data capture tools hosted at Ruhr University Bochum.

### 2.4. Definitions

All EMS protocols include a mandatory field for the working diagnosis. Protocols were systematically screened to identify the primary diagnostic suspicion documented by EMS personnel. The primary endpoint was considered met if pulmonary embolism was recorded as the main diagnosis. To account for potential documentation or transmission errors, protocols were additionally screened for any mention of suspected pulmonary embolism, including instances where it was documented only as a secondary diagnosis; in these cases, the primary endpoint was also considered met. Where risk scoring instruments (NEWS2 score, PESI, and sPESI) had not already been calculated by EMS personnel or the emergency department, these scores were retrospectively reconstructed based on the available documented parameters. Cases with missing individual parameters required for score calculation were not excluded from the score analysis, resulting in a potential underestimation.

Patients were classified according to the 2019 ESC Guidelines [7]. Risk stratification was performed at ED admission, as this was the first time point at which all required parameters were available. High-risk PE was defined by hemodynamic instability, i.e., cardiac arrest requiring cardiopulmonary resuscitation, obstructive shock, or persistent hypotension. As the duration of hypotension and signs of end-organ hypoperfusion could not be reliably assessed from retrospective records, two documented systolic blood pressure measurements < 90 mmHg documented by EMS were considered sufficient. In hemodynamically stable patients, intermediate-high risk was assigned in the presence of PESI class III–V or sPESI ≥ 1 together with both RV dysfunction on CTPA and elevated cardiac troponin at ED admission; intermediate-low risk if none or only one of these criteria was present; and low risk if PESI class I–II or sPESI 0 without RV dysfunction or elevated troponin.

Based on the primary endpoint, patients were categorized into two groups: those with correct prehospital detection of pulmonary embolism (“detected”) and those without (“not detected”). This classification was used for subsequent comparative analyses of secondary outcomes and treatment characteristics.

Hypoxemia was defined as peripheral oxygen saturation < 90%. Tachycardia was defined as a heart rate above 100 beats per minute. Hypotension was defined as systolic blood pressure < 100 mmHg.

The time point of pulmonary embolism diagnosis was defined as the time of CTPA acquisition. Accordingly, time to diagnosis was calculated as the interval from the EMS alarm time to the time of CTPA acquisition.

To describe diagnostic timing in the emergency department, we categorized CTPA acquisition as occurring within 6 h or after 6 h of ED admission. We therefore additionally calculated the time interval from ED admission to CTPA acquisition.

### 2.5. Endpoints

The primary endpoint was the diagnostic sensitivity of prehospital detection of pulmonary embolism by emergency medical services. Diagnostic sensitivity was defined as the proportion of patients with CTPA-confirmed (diagnostic confirmation) central or multisegmental PE in whom PE was documented as the main or secondary working diagnosis in the EMS protocol. As only patients with confirmed PE were included, specificity and predictive values could not be determined. Secondary endpoints included time to definitive diagnosis (from EMS alarm to confirmatory CTPA), in-hospital mortality, need for cardiopulmonary resuscitation, admission to the intensive care unit or intermediate care unit, length of ICU stay (admission time ICU to discharge from ICU), treatment modality, and bleeding complications. Additionally, the association between characteristics of prehospital EMS care, including physician-staffed response and prehospital initiation of anticoagulation and clinical outcomes was assessed. Death was classified as PE-related only if pulmonary embolism had been confirmed using CTPA, and the in-hospital documentation established obstructive shock or cardiac arrest attributable to pulmonary embolism as the cause of death. Attribution was based on the complete in-hospital records, including intensive care documentation and discharge letters. Bleeding events during the index hospitalization were reviewed according to the ISTH criteria [29] only for major bleeding, if patients did not meet these criteria and the records did not consistently allow further differentiation, events are reported as documented bleeding events without severity classification.

### 2.6. Statistical Analyses

Continuous variables are presented as median (interquartile range, IQR) or mean (standard deviation, SD), depending on distribution. Categorical variables are presented as absolute numbers and percentages, n (%). Diagnostic sensitivity was calculated as the proportion of patients with correct prehospital recognition of pulmonary embolism, with 95% confidence intervals derived using the Wilson score method. Subgroup comparisons of categorical variables were performed using Fisher’s exact test, given the small sample sizes in several subgroups. Secondary outcomes, treatment characteristics, and complications were compared between patients with and without prehospital PE recognition using Fisher’s exact test for categorical variables and the Wilcoxon rank-sum test for continuous variables. Paired comparisons of vital signs between EMS arrival and handover were performed using the Wilcoxon signed-rank test. Time to diagnosis was analyzed using the Kaplan–Meier method, and differences between groups were assessed using the log-rank test. Multivariable logistic regression was used to assess the association between prehospital anticoagulation and binary clinical outcomes (ICU admission and in-hospital mortality), adjusted for age and NEWS2 score at EMS arrival. The association with length of ICU stay was assessed using multivariable linear regression on log-transformed values, given the right-skewed distribution of this variable; regression coefficients were back-transformed and are reported as percentage change. As early death may influence ICU duration analyses, sensitivity analysis restricted to survivors was performed.

Prehospital predictors of time to CTPA acquisition were assessed using multivariable Cox proportional hazards regression. Event definition: the event was defined as CTPA acquisition; as all included patients underwent CTPA, every patient contributed an event and no censoring occurred. Time origin: time was measured from EMS activation. Exclusions: patients with missing or implausible EMS activation timestamps were excluded. Covariate selection: covariates were selected a priori based on clinical plausibility and their availability during prehospital care, rather than by data-driven selection, and comprised age, sex, physician-staffed EMS response, hypoxemia (SpO_2_ < 90%), tachycardia (heart rate > 100/min), oxygen administration, and prehospital intravenous heparin. Proportional hazards assessment: the proportional hazards assumption was assessed using Schoenfeld residuals; a hazard ratio above 1 indicates a higher instantaneous rate of reaching the diagnostic endpoint. For covariates violating the proportional hazards assumption, sensitivity analyses were performed using a Cox model stratified by the respective covariate and an accelerated failure time model with a log-normal distribution, with results reported as time ratios. This analysis was exploratory.

Subgroup analyses of diagnostic sensitivity were exploratory in nature, and no correction for multiple testing was applied. A two-sided *p*-value < 0.05 was considered statistically significant. All statistical analyses were performed using R (version 4.5, R Foundation for Statistical Computing, Vienna, Austria). The AI language model Claude (Anthropic, claude-sonnet-4-6) was used to assist with R code formatting as well as the revision of data visualizations. All outputs were reviewed and edited by the authors, who take full responsibility for the content of this publication.

## 3. Results

Between 1 January 2019 and 31 December 2024, a total of 735 patients with a diagnosis of pulmonary embolism were screened for eligibility. Most cases (n = 483) were excluded because pulmonary embolism was not the primary diagnosis, including cases of hospital-acquired pulmonary embolism and inter-hospital transfers. After further exclusions, a final cohort of 171 patients with complete prehospital emergency medical services documentation was included in the analysis (Figure 1). The demographic data are presented in Table 1. Median patient age was 73 years (IQR 62–82), and sex distribution was balanced (52% male). The median PESI score was 101 (IQR 84–122), with most patients classified as intermediate-high-risk (29.8%) or high-risk (26.3%) according to the ESC risk classification. ESC risk class had been documented in the medical records in 138 patients and was calculated retrospectively in 33 patients. Of the 45 high-risk patients, 40 were classified based on documented cardiac arrest or obstructive shock and 5 retrospectively based on two systolic blood pressure values <90 mmHg at EMS arrival and handover. Baseline medication and comorbidities are reported in Table A1 and Table A2 in Appendix A.

### 3.1. Diagnostic Sensitivity for Pulmonary Embolism

**Figure 2:**
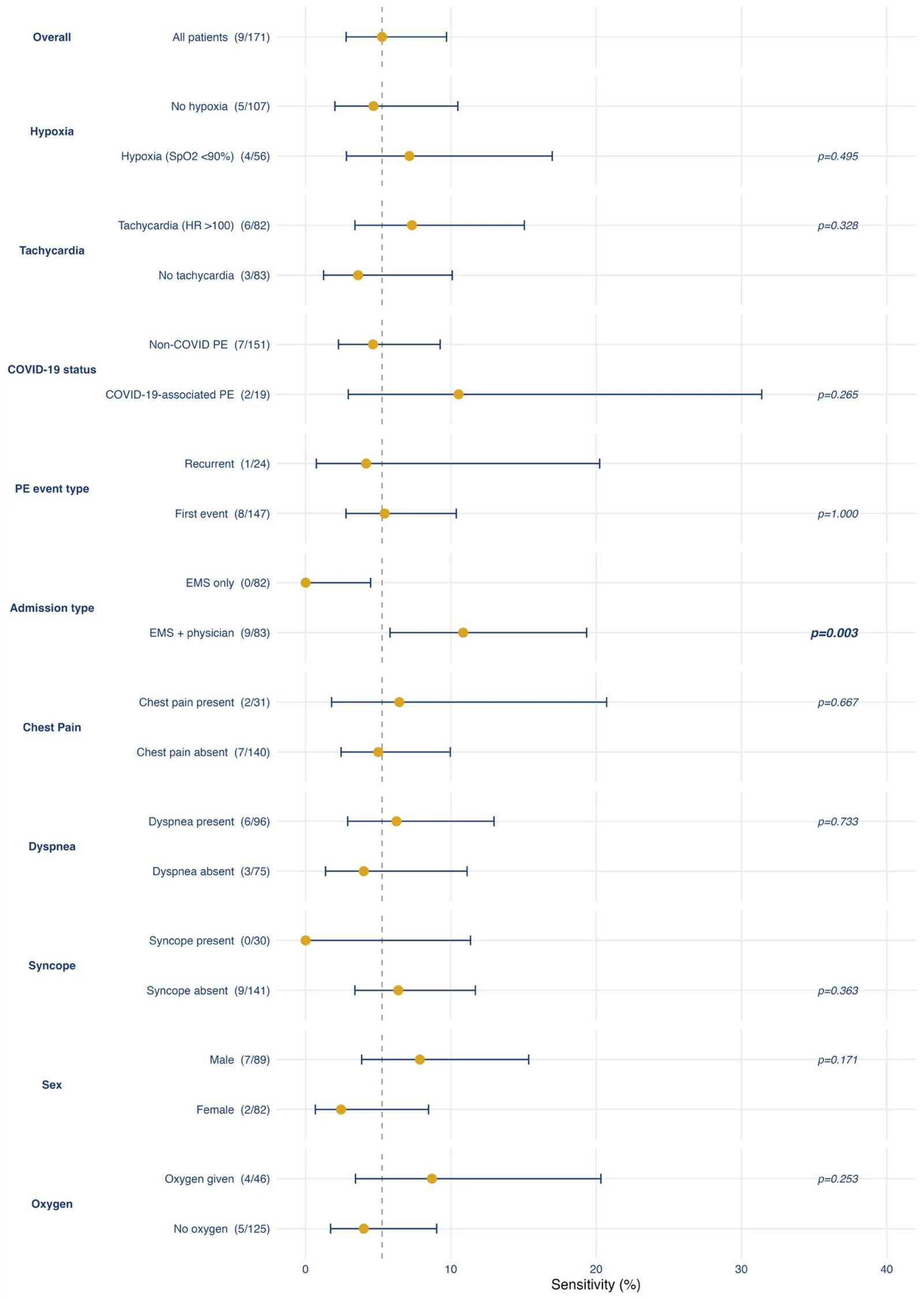
Prehospital diagnostic sensitivity for pulmonary embolism overall and across prespecified subgroups. The dashed vertical line represents the overall sensitivity estimate (5.3%, 95% CI 2.8–9.7%). Dots represent the point estimate of diagnostic sensitivity within each subgroup, with horizontal bars indicating the corresponding 95% confidence intervals. The dashed vertical line denotes the sensitivity of the overall cohort (primary endpoint) as a reference. *p*-values were derived from Fisher’s exact test. Subgroup analyses were exploratory; no adjustment for multiple testing was performed. The admission type subgroup (bold) was the only significant predictor of prehospital PE recognition (*p* = 0.003). CI, confidence interval; EMS, emergency medical services; PE, pulmonary embolism; SpO_2_, peripheral oxygen saturation.

The prehospital diagnostic sensitivity for pulmonary embolism was 5.3% (95% CI, 2.8–9.7%), with pulmonary embolism correctly detected in 9 of 171 patients. Among the 162 patients in whom PE was not detected, the most frequent alternative EMS working diagnoses were unclear dyspnea/respiratory distress (n = 20, 11.7%), pneumonia (n = 18, 10.5%), and myocardial infarction (n = 29, 17%) (Table 2). Subgroup analysis revealed a significant difference in prehospital diagnostic sensitivity according to the mode of EMS response: all correctly detected cases occurred when an emergency physician was present (EMS with physician: 10.8% vs. EMS only: 0%, *p* = 0.003). Further subgroup analyses showed no significant differences in sensitivity according to sex, symptoms, oxygen administration, hemodynamics and PE-event type (Figure 2).

### 3.2. Symptoms

**Figure 3:**
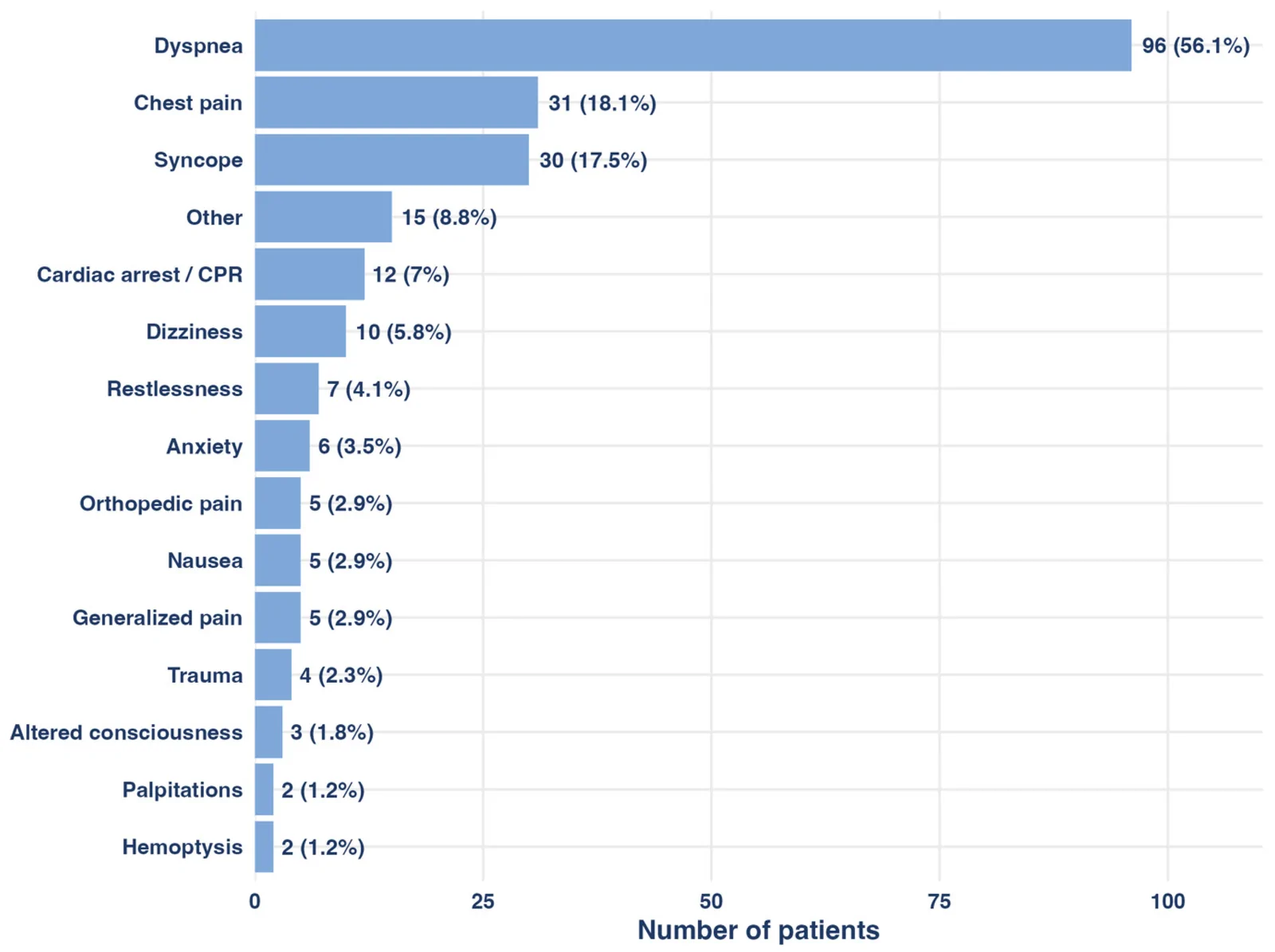
Frequency of leading symptoms at the time of emergency medical services contact. Bars represent the number of patients with each documented leading symptom. CPR, cardiopulmonary resuscitation.

Symptom onset coincided with the EMS call in most patients (69%), whereas in 31% of patients, symptoms had preceded the EMS call by a median of 3 days (IQR 1.5–7 days). The most frequent chief complaints were dyspnea (56.1%), chest pain (18.1%), and syncope (17.5%) (Figure 3). Hemoptysis was present in 2 of 171 patients (1.2%).

### 3.3. Prehospital Hemodynamics and Treatment

**Figure 4:**
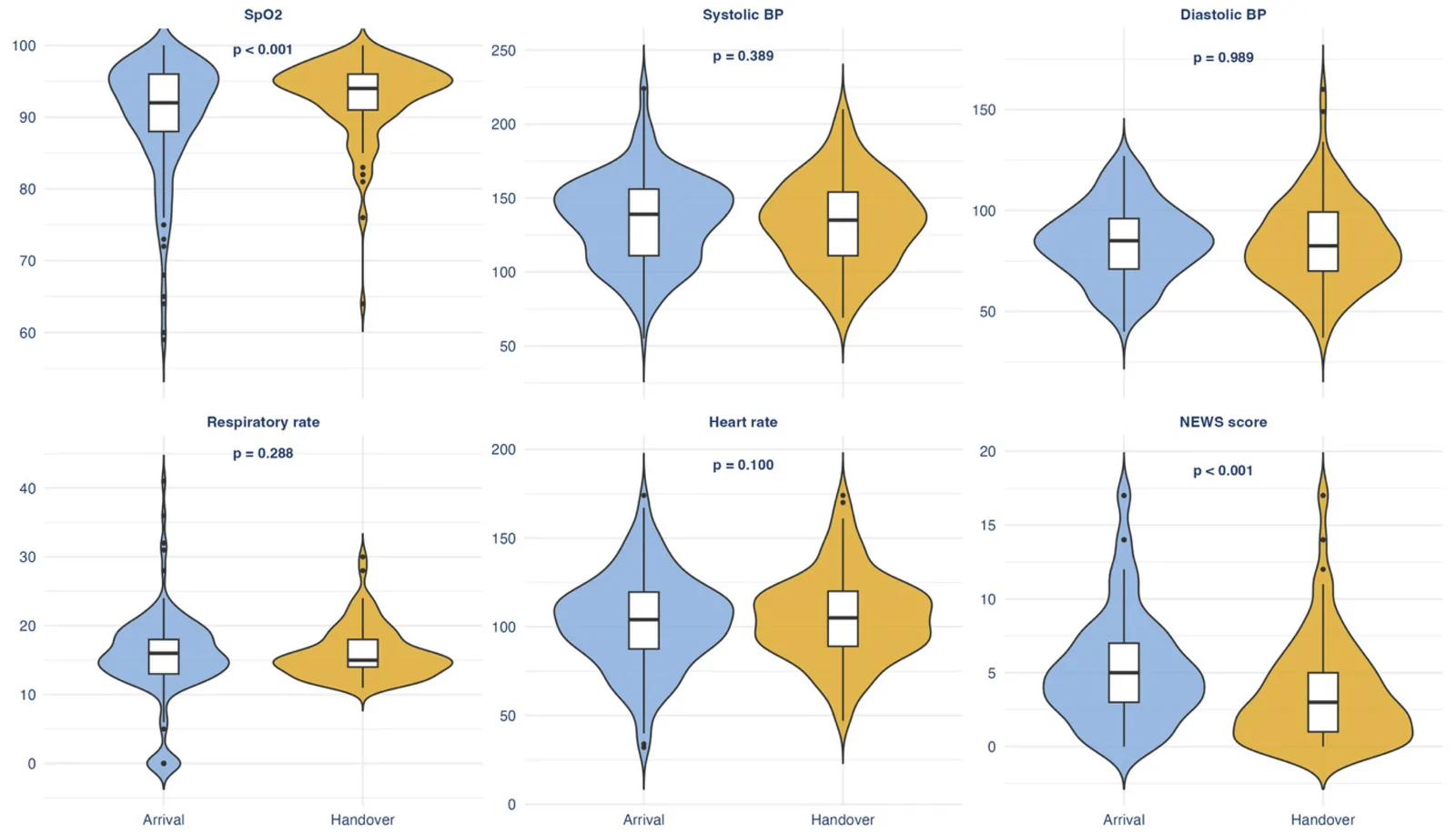
Vital signs at EMS arrival and handover to the emergency department. Violin plots show the distribution of key vital parameters at EMS arrival (blue) and at handover to the emergency department (yellow). Horizontal lines within each violin indicate the median. Individual dots represent outliers, defined as values beyond 1.5 × the interquartile range from the first or third quartile. *p*-values were derived from the Wilcoxon signed-rank test for paired comparisons between arrival and handover. HR, heart rate; NEWS, National Early Warning Score; BP, blood pressure in mmHg; SpO_2_, peripheral oxygen saturation in %; respiratory rate in/min.

At EMS arrival, patients were tachycardic, with a median heart rate of 104 beats per minute (IQR 86–120), and normotensive, with a median systolic blood pressure of 139 mmHg (IQR 111–156) and diastolic blood pressure of 85 mmHg (IQR 71–96). Peripheral oxygen saturation (SpO_2_) was reduced at a median of 92% (IQR 88–96) and increased significantly during EMS treatment to 94% (IQR 91–96, *p* < 0.001). Median NEWS2 score on arrival was 5 (IQR 3–7) and decreased during EMS treatment to 3 (IQR 1–5, *p* < 0.001) (Figure 4).

Prehospital treatment was administered in 37.4% of patients, including oxygen in 46 patients (26.9%) and intravenous heparin in 28 patients (16.4%). Of the heparin-treated patients, 4 belonged to the PE-detected group (4/9, 44.4%) and 24 to the not-detected group (24/162, 14.8%). In all 24 heparin-treated patients without prehospital PE detection, heparin was administered for suspected myocardial infarction. No cases of prehospital thrombolysis were performed. A total of 14 patients (8.2%) required intubation and mechanical ventilation, while non-invasive ventilation was performed in one patient (0.6%). A total of 17 patients (9.9%) required hemodynamic stabilization with catecholamines or vasopressors.

### 3.4. Time to Diagnosis

**Figure 5:**
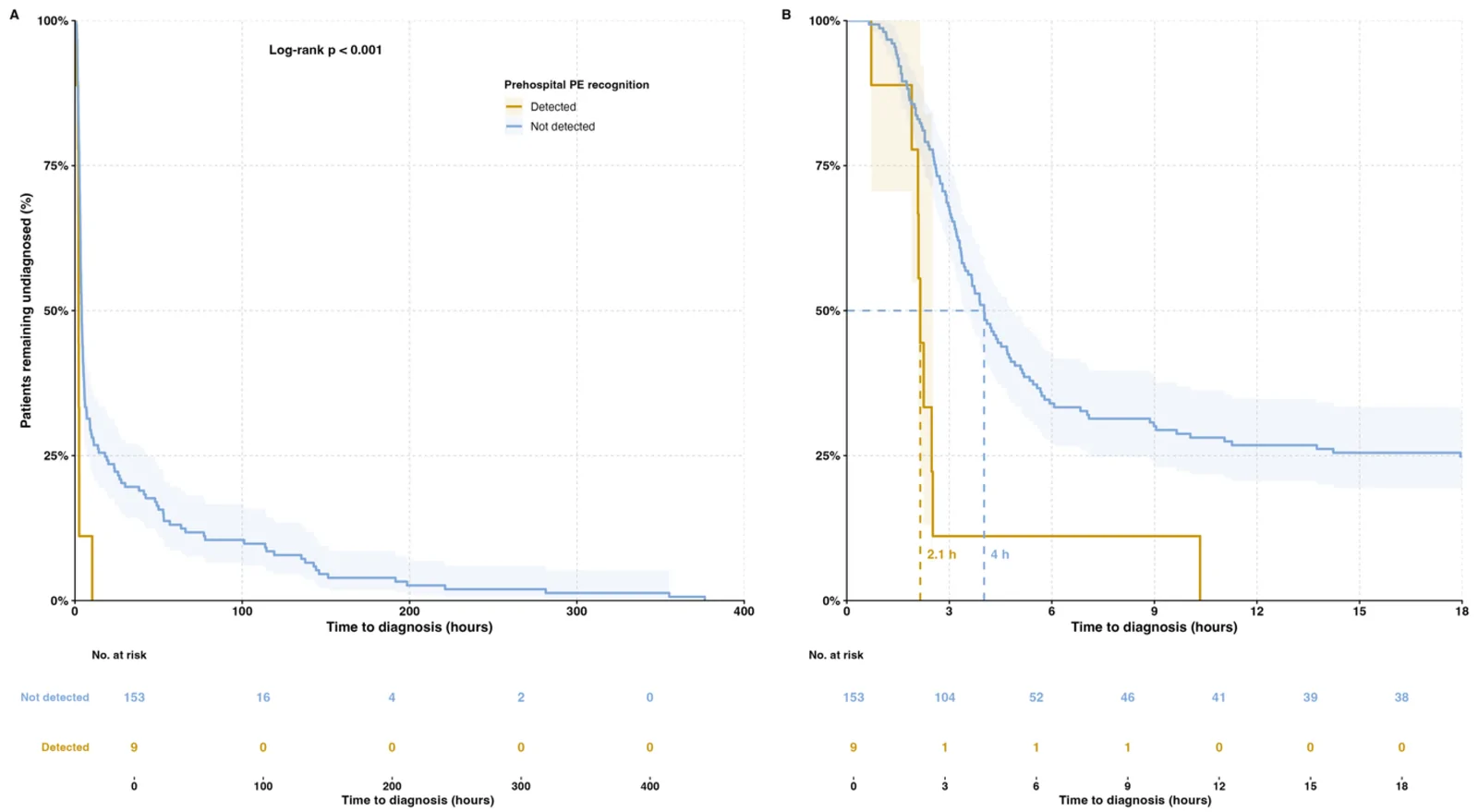
Kaplan–Meier analysis of time to definitive diagnosis stratified by prehospital pulmonary embolism recognition status. Panel (**A**) shows the full observation period; panel (**B**) displays a zoomed view restricted to the first 18 h after EMS alarm. Time to diagnosis was defined as the interval from EMS alarm to computed tomography pulmonary angiography acquisition. Patients with correct prehospital PE detection (Detected, n = 9) are compared with those without prehospital detection (Not detected, n = 153). Dashed horizontal and vertical lines indicate median time to diagnosis per group (Detected: 2.1 h; Not detected: 4.0 h). The *p*-value was derived from the log-rank test. CTPA, computed tomography pulmonary angiography; EMS, emergency medical services; PE, pulmonary embolism. Nine patients with missing or implausible EMS alarm timestamps were excluded.

The median time to diagnosis from EMS call to final CT confirmation was 3.9 h (IQR 2.5–16.1) across all patients. Time to diagnosis was significantly shorter in patients with correct prehospital PE detection (median 2.1 h, IQR 2.1–2.5) compared to those without early recognition (median 4.0 h, IQR 2.6–18.0; log-rank *p* < 0.001) (Figure 5). The maximum time to diagnosis was 10 h among detected cases, compared with 376 h (15.7 days) among not detected cases.

In a multivariable Cox regression model restricted to prehospital variables, prehospital initiation of anticoagulation was the only significant independent predictor of higher rate of earlier diagnosis (HR 2.42, 95% CI 1.40–4.19, *p* = 0.002). Tachycardia and oxygen administration were not significantly associated with time to diagnosis (Table 3). The proportional hazards assumption was met for prehospital anticoagulation (*p* = 0.145). The association of prehospital anticoagulation was consistent in sensitivity analyses not relying on the proportional hazards assumption (stratified HR 2.31, 95% CI 1.34–3.98; accelerated failure time model, time ratio 0.39, 95% CI 0.19–0.79). The proportional hazards assumption was violated for hypoxemia (*p* < 0.001) and for physician-staffed EMS response (*p* = 0.048), suggesting time-varying effects for these variables; their estimates should therefore be interpreted as averaged effects (Table A3 in Appendix A).

While patients with correct prehospital PE detection were numerically more likely to be diagnosed within 6 h of ED presentation (88.9% vs. 65.6%), this difference did not reach statistical significance (OR 4.16, 95% CI 0.54–189.1, *p* = 0.274). Of 169 patients with available timestamps (two patients were excluded due to missing data), 56 (33.1%) were not diagnosed with PE within 6 h of ED presentation. Of these, 38 patients (22.5% of patients with available timestamps) were diagnosed only after more than 24 h. No patient with delayed diagnostic confirmation was excluded because of uncertainty regarding attribution to the index EMS encounter.

### 3.5. Treatment, Mortality and Complications

Overall, 25 patients died (14.6%), all of whom had pulmonary embolism-related deaths. CPR was performed in 18 patients (10.8%), of whom 12 (7.5%) had already been resuscitated by EMS. Regarding level of care, 31% of patients were admitted to the general ward, and 40.5% were admitted to the intensive care unit or intermediate care unit (28.6%), with no significant differences according to early detection (Table 4). Of the 68 patients admitted to the ICU, 68 were admitted directly to the ICU and 17 were transferred to the ICU during the course of hospitalization after initial admission to the general ward or IMC. Among these 85 patients, the median ICU duration was 40.4 h (IQR 28.0–43.6) in patients with prehospital PE recognition (n = 6) and 39.3 h (IQR 19.4–90.3) in those without (n = 79; *p* = 0.75). Seventy-eight patients (45.6%) had signs of right heart strain on computed tomography, with no significant difference between both groups (55.6% vs. 45.6%, *p* = 0.7).

Catheter-directed thrombolysis (CDT) was performed in 16 patients (11.6%), systemic thrombolysis in 19 patients (13.8%), and most patients (102, 73.9%) received anticoagulation only, with no significant differences across subgroups. Bleeding complications occurred in 14 patients (8.6%); none met the ISTH criteria for major bleeding, and no further differentiation between clinically relevant non-major and minor bleeding was performed (Table 5).

### 3.6. Association of Prehospital Treatment with Clinical Outcomes

In a multivariable analysis adjusted for age and NEWS2 score, prehospital initiation of anticoagulation was not associated with shorter ICU stay (13.2%, 95% CI −54.4–180.9, *p* = 0.79), but prehospital anticoagulation was associated with a higher likelihood of ICU admission (OR 3.66, 95% CI 1.39–10.91, *p* = 0.008), while no significant association was found regarding mortality (OR 1.51, 95% CI 0.44–5.23, *p* = 0.46). Older age was associated with a shorter ICU duration (−3.4% per year, 95% CI −5.9 to −0.9, *p* = 0.01), which was consistent when the analysis was restricted to survivors (−2.6% per year, 95% CI −4.4% to −0.7%). NEWS2 score was independently associated with ICU admission (OR 1.26 per point, *p* < 0.001) and mortality (OR 1.36 per point, *p* < 0.001) across all models (Table 6).

## 4. Discussion

The main findings of this observational study on prehospital PE detection are as follows: (1) the diagnostic sensitivity for PE detection by EMS was critically low at 5.3%; (2) time to definitive diagnosis was significantly shorter in patients with correct prehospital PE detection compared to those without (median 2.1 h vs. 4.0 h, log-rank *p* < 0.001); (3) a clinically relevant proportion of patients experienced substantial diagnostic delay, with 38 patients (22.5%) receiving a diagnosis more than 24 h after ED presentation; and (4) prehospital initiation of anticoagulation was independently associated with a 2.4-fold higher rate of earlier definitive diagnosis. In the primary analysis, prehospital heparin was associated with a shorter ICU stay; however, this association was no longer statistically significant in a sensitivity analysis restricted to survivors.

To our knowledge, this is the first study to investigate the diagnostic sensitivity of pulmonary embolism suspicion by EMS staffed with and without emergency physicians. Only 9 of 171 patients were correctly identified as having a pulmonary embolism in the prehospital setting, yielding a sensitivity of only 5.3%. Of note, only patients with confirmed PE were included. This estimate reflects documented prehospital suspicion conditional on subsequent confirmation, and specificity and predictive values could not be determined. As only central or multisegmental PE were included, the real prehospital detection rate of PE may presumably be lower. Notably, all correctly identified cases were diagnosed by physician-staffed EMS units. To date, data on the diagnostic sensitivity of prehospital pulmonary embolism detection remain limited. Rouleau et al. demonstrated that 21.5% of patients with PE presenting to the emergency department via EMS had a lower frequency of discharge and higher 30-day all-cause mortality [30].

Meta-analyses have shown that clinical gestalt, i.e., suspicion of PE based on clinical judgment alone, without decision tools, laboratory testing, or imaging, can reach sensitivities of 85% [22] and 74% [23], respectively. All these studies were conducted in the emergency department, with emergency physicians assessing patients on site, and were mostly intended to safely rule out PE rather than rule in PE.

The overall low sensitivity for PE may be due to highly variable PE symptoms, yielding misdiagnoses in up to 27.5% of PE cases [26]. In addition to dyspnea and chest pain, syncope was the third leading symptom (17.5%) among hospital admissions with undiagnosed PE. A previous study reported a prevalence of PE of 17.3% among patients hospitalized for syncope [16]. However, larger registry studies showed that the prevalence of PE in large syncope populations was low [17]. Hemoptysis is often considered a classic PE symptom, though its real-world prevalence is low—1.2% in our study, consistent with previous data [11].

As a contributing factor, EMS providers have less time to evaluate, diagnose, and transport patients to the hospital, whereas the diagnostic work-up in the ED, after initial stabilization, may allow more time to sufficiently assess patients. PE scoring tools were reported in only 3 of 171 patients, which may support the suspicion that PE was not considered in the differential diagnosis. Furthermore, PE scoring tools are not established in the standard operating procedures (SOPs) for the management of dyspnea in the Bochum EMS region, nor are they included in the current guidelines [7,15].

Advanced diagnostic work-up is limited in the EMS setting: apart from vital signs, physical examination, and clinical history, no further diagnostics such as D-dimer testing, echocardiography, or blood gas analysis were available between 2019 and 2024. It should be noted, however, that current guidelines do not recommend transthoracic echocardiography (TTE) as a stand-alone diagnostic tool for diagnosing PE. TTE should only be performed at the bedside in hemodynamically unstable patients with suspected high-risk PE to detect signs of right heart strain and guide immediate treatment decisions, whereas in hemodynamically stable patients, CTPA should be performed directly as the diagnostic test of choice [7]. However, early TTE has additionally been shown to be of prognostic value, predicting short-term mortality in patients with confirmed PE [31,32]. As point-of-care ultrasound (POCUS) is increasingly being rolled out for widespread implementation in EMS, point-of-care TTE (POC-TTE) may help detect PE in the prehospital setting, as case series have already suggested, although controlled data are still lacking [33].

Our data show that a correct prehospital diagnosis of PE was associated with less time to diagnosis, with all patients receiving CTPA within the first 10 h after the EMS call. In addition, 162 patients were admitted with other working diagnoses, mostly suspected myocardial infarction, pneumonia, or dyspnea of unclear origin. Of these 162 patients, 113 were subsequently correctly identified as having pulmonary embolism within the first 6 h of ED admission. According to our definition of delayed diagnostic confirmation as CTPA acquisition more than 6 h after ED admission, diagnostic confirmation was delayed in 33.1% of patients, which may, in part, reflect logistical factors such as limited CT availability, clinical stabilization, or competing diagnostic priorities rather than a true diagnostic delay. However, 22.5% of patients were diagnosed later than 24 h after admission, a delay that can hardly be explained by logistical factors alone and aligns with previously reported misdiagnosis rates of approximately 27.5% [26].

A total of 25 patients died (14.6%), which is consistent with previous mortality data [34]. No difference in mortality was found according to correct prehospital PE suspicion, most likely because the overall PE detection rate was low and the study was not powered to evaluate mortality outcomes. Mortality may be underestimated, as a final diagnosis of PE was only established in patients with a positive CTPA; patients undergoing ongoing CPR therefore needed to achieve at least a short-term return of spontaneous circulation (ROSC) to be included in the study. In cases of out-of-hospital cardiac arrest (OHCA), mortality from PE rises to up to 65% [34]. There were also no differences in ICU admission rates or ICU length of stay according to correct PE diagnosis.

According to the ESC risk classification, 39 patients (22.8%) were classified as low-risk, 36 (21.1%) as intermediate-low-risk, 51 (29.8%) as intermediate-high-risk, and 45 (26.3%) as high-risk. The high proportion of high-risk cases compared to other studies [11] likely reflects a selection bias inherent to EMS-transported cohorts, in which hemodynamically unstable patients may be overrepresented compared with the general PE population [30]. Owing to the retrospective design, persistent hypotension could not be confirmed according to the ESC definition in five patients. In these patients, the required duration of at least 15 min was approximated by systolic blood pressure values < 90 mmHg despite treatment at both EMS arrival and ED arrival, which was considered to fulfill the ESC criterion. This may have led to an overestimation of the high-risk cohort in these five patients, and this classification should be interpreted with caution.

During EMS transport, the NEWS2 score improved from 5 to 3, indicating that patients stabilized during prehospital care. The NEWS2 score was originally validated by the Royal College of Physicians to evaluate the severity of acute illness [35]; thresholds above 5 indicate medium risk, while scores above 7 indicate high risk. NEWS2 was recently used by Rosenfield et al. as part of the primary endpoint in the HI-PEITHO trial [14] to detect and objectify hemodynamic collapse (NEWS2 > 9). Our data show that patients were at medium risk at EMS arrival and stabilized during care, and that the NEWS2 score was independently associated with ICU admission and mortality. Of note, NEWS2 was assessed retrospectively rather than prospectively reported by EMS.

Most patients received conservative treatment with anticoagulation alone, whereas catheter-directed thrombolysis (CDT) was performed in 11.6% of patients and systemic thrombolysis in 13.8%. Given that the study period spanned 2019–2024, interventional therapies were not yet as widely established as they are today [12]. Considering that 29.8% of patients were classified in the ESC intermediate-high-risk group, a considerably higher rate of CDT would be expected today.

Our data demonstrate that prehospital treatment initiation with intravenous anticoagulation was associated with a significantly (2.4-fold) higher rate of earlier diagnosis; a statistically significant association with mortality was not detected. One possible explanation is that a relevant proportion of patients in the not detected cohort were admitted with suspected myocardial infarction and consequently received prehospital anticoagulation, which may have triggered an expedited diagnostic pathway and thereby facilitated earlier PE diagnosis. The associations with earlier diagnosis are therefore presumably attributable to the accelerated diagnostic process rather than to anticoagulation itself. Nonetheless, these patients represent an interesting subgroup characterized by faster diagnostic confirmation. Furthermore, these data should be interpreted with caution, as the study was not adequately powered to evaluate these endpoints and should therefore be considered exploratory only.

For the first time, we present systematic data demonstrating the underdiagnosis of pulmonary embolism in the prehospital EMS setting and that documented prehospital PE suspicion was associated with earlier confirmatory CTPA. These findings may serve as the basis for the design of prospective trials aimed at improving prehospital PE recognition.

### Limitations

This study has several limitations. First, the retrospective design relies on the completeness and accuracy of documentation in prehospital protocols, which may have led to underreporting of clinical findings or reasoning that influenced diagnostic decision-making but was not recorded. Second, the study was conducted at a single center, which may limit the generalizability of our findings to EMS systems with different staffing models, training standards, or resource availability, particularly regarding the use of physician-staffed versus paramedic-only teams. The relatively small number of correctly identified and confirmed PE cases (n = 9) limits the statistical power of subgroup analyses and increases the risk of type II error, so these findings should be interpreted as hypothesis-generating rather than confirmatory. The small number of recognized cases precluded reliable conclusions on clinical outcomes. Associations involving prehospital anticoagulation remain exploratory and may reflect differences in treatment indication and care pathways. Although all cases with markedly delayed diagnosis were reviewed by two investigators and agreed upon by consensus, minor misclassification in the attribution of PE to the index EMS encounter cannot be entirely excluded.

## 5. Conclusions

In this single-center cohort of EMS-transported patients with subsequently confirmed central or multisegmental pulmonary embolism, prehospital PE suspicion was documented in only 5.3% of cases and was associated with earlier imaging. The small number of recognized cases precluded reliable conclusions on clinical outcomes; however, a relevant number of patients experienced a substantial delay in the correct pulmonary embolism diagnosis. These findings highlight the urgent need for EMS-specific diagnostic strategies and prospective trials to improve prehospital PE detection.

## Figures and Tables

**Table 1:** Baseline characteristics.

Characteristic	N = 171 ^1^
Age, years, median (IQR)	73 (62, 82)
Male sex	89 (52.0%)
COVID-19-associated PE	19 (11.2%)
Recurrent PE event	24 (14.0%)
Risk scoring	
PESI score, median (IQR) ^a^	101 (84, 122)
ESC risk class: ^b^	
Low-risk	39 (22.8%)
Intermediate-low-risk	36 (21.1%)
Intermediate-high-risk	51 (29.8%)
High-risk	45 (26.3%)
Cardiovascular risk profile	
Diabetes mellitus	24 (14.0%)
Hypercholesterolemia	21 (12.3%)
Obesity	28 (16.4%)
Smoking (current/former)	17 (9.9%)
Arterial hypertension	74 (43.3%)
Family history of cardiovascular disease	2 (1.2%)

Continuous variables are presented as median (IQR); ^1^ categorical variables as n (%). ^a^ At least one score component was undocumented and assigned zero points in 20 of 171 patients for PESI. ^b^ ESC risk class was documented in the medical records in 138 patients and calculated retrospectively in the remaining 33 patients, without missing data. Of these 33 patients, 5 were classified as high risk, 8 as intermediate-low risk, and 20 as low risk. ESC, European Society of Cardiology; IQR, interquartile range; PE, pulmonary embolism; PESI, Pulmonary Embolism Severity Index.

**Table 2:** Prehospital EMS working diagnoses at hospital presentation.

Characteristic	N = 171 ^1^
COPD/asthma exacerbation	4 (2.3%)
Cardiac arrhythmia	6 (3.5%)
Heart failure/pulmonary edema	4 (2.3%)
Infection	6 (3.5%)
Pulmonary embolism	9 (5.3%)
Myocardial infarction	29 (17.0%)
Neurological diagnosis	6 (3.5%)
Other	32 (18.7%)
Other cardiovascular disease	8 (4.7%)
Pneumonia	18 (10.5%)
Syncope	18 (10.5%)
Trauma	11 (6.4%)
Unclear dyspnea/respiratory distress	20 (11.7%)

^1^ n (%).

**Table 3:** Multivariable Cox regression analysis of prehospital predictors of time to definitive diagnosis.

Characteristic	n/N (%) or Median (IQR)	HR	95% CI	*p*-Value
Age, per year	72 (62–82)	1.00	0.99, 1.01	0.6
Male sex	77/144 (53.5%)	1.16	0.81, 1.65	0.4
EMS + physician response	69/144 (47.9%)	1.37	0.95, 1.99	0.10
Hypoxemia (SpO_2_ < 90%)	49/144 (34.0%)	0.75	0.49, 1.14	0.2
Tachycardia (HR > 100/min)	74/144 (51.4%)	0.83	0.58, 1.17	0.3
Oxygen administration	37/144 (25.7%)	1.07	0.71, 1.62	0.7
Prehospital anticoagulation	21/144 (14.6%)	2.42	1.40, 4.19	0.002

Exploratory analysis. Time origin was EMS activation and the event was CTPA acquisition; as all patients underwent CTPA, no censoring occurred. Of 171 patients, 9 were excluded owing to missing or implausible EMS activation timestamps and 18 owing to missing covariate data (heart rate missing, n = 3; implausible data, n = 3; SpO_2_ and heart rate missing, n = 3; SpO_2_ missing, n = 9), yielding 144 patients with 144 events. The hazard ratio for age refers to a one-year increase. A hazard ratio (HR) above 1 indicates a higher instantaneous rate of reaching the diagnostic endpoint. The proportional hazards assumption was violated for hypoxemia (*p* < 0.001) and physician-staffed EMS response (*p* = 0.048). CI, confidence interval; EMS, emergency medical services; HR, hazard ratio; SpO_2_, peripheral oxygen saturation.

**Table 4:** Secondary clinical outcomes by prehospital pulmonary embolism detection status.

Characteristic	Overall N = 171 ^1^	Detected N = 9 ^1^	Not Detected N = 162 ^1^	Effect Estimate (95% CI)	*p*-Value
CPR performed (overall) ^a^	18/166 (10.8%)	1/7 (14.3%)	17/159 (10.7%)	1.39 (0.03–12.57)	0.6
Prehospital (EMS) ^b^	12/161 (7.5%)	1 (11.1%)	11/152 (7.2%)	1.60 (0.03–13.96)	0.5
In-hospital mortality	25 (14.6%)	1 (11.1%)	24 (14.8%)	0.72 (0.02–5.80)	>0.9
Initial type of admission ^c^					0.065
General ward	52/168 (31.0%)	0 (0.0%)	52/159 (32.7%)	0.00 (0.00–1.09)	
Intensive care unit	68/168 (40.5%)	6 (66.7%)	62/159 (39.0%)	3.11 (0.64–19.90)	
Intermediate care unit	48/168 (28.6%)	3 (33.3%)	45/159 (28.3%)	1.26 (0.20–6.23)	
ICU duration, hours	39.5 (20.3–80.9)	40.4 (28.0–43.6)	39.5 (20.0, 88.8)	−4.1 (−50.9 to 21.9)	0.75
Right heart strain on CT ^d^	78/169 (46.2%)	5 (55.6%)	73/160 (45.6%)	1.49 (0.31–7.78)	0.7

Data are presented as ^1^ n/N (%) for categorical variables and as median (interquartile range) for continuous variables. The *p*-values were derived from Fisher’s exact test for categorical variables and the Wilcoxon rank-sum test for continuous variables. Effect estimates compare patients with versus without prehospital PE recognition and are reported as odds ratios with exact 95% confidence intervals (Fisher’s exact test) for categorical variables (for initial level of care, each category versus all others) and as Hodges–Lehmann median differences with 95% confidence intervals for ICU duration. ^a^ Missing in 5 of 171 patients. ^b^ Missing in 10 of 171 patients. ^c^ Missing in 3 of 171 patients. ^d^ Missing in 2 of 171 patients. CPR, cardiopulmonary resuscitation; ICU, intensive care unit; EMS, emergency medical services; CT, computed tomography.

**Table 5:** In-hospital treatment modalities, anticoagulation, and bleeding complications by prehospital pulmonary embolism detection status.

Characteristic	Overall N = 171 ^1^	Detected N = 9 ^1^	Not Detected N = 162 ^1^	Effect Estimate (95% CI)	*p*-Value
Treatment modality ^a^					0.13
Systemic thrombolysis	19/138 (13.8%)	2/7 (28.6%)	17/131 (13.0%)	2.66 (0.24–17.89)	
Conservative (anticoagulation only)	102/138 (73.9%)	3/7 (42.9%)	99/131 (75.6%)	0.25 (0.03–1.53)	
Interventional thrombectomy	1/138 (0.7%)	0/7 (0.0%)	1/131 (0.8%)	0.0 (0.0–721.64)	
Catheter-directed thrombolysis	16/138 (11.6%)	2/7 (28.6%)	14/131 (10.7%)	3.30 (0.29–22.65)	
Initial anticoagulation route ^b^					0.4
Direct oral anticoagulant (DOAC)	48/128 (37.5%)	2/7 (28.6%)	46/121 (38.0%)	0.65 (0.06–4.20)	
Intravenous heparin	58/128 (45.3%)	5/7 (71.4%)	53/121 (43.8%)	3.18 (0.50–34.63)	
Subcutaneous heparin	22/128 (17.2%)	0/7 (0.0%)	22/121 (18.2%)	0.00 (0.00–3.38)	
Bleeding complications					>0.9
No documented bleeding events	157/171 (91.8%)	9/9 (100.0%)	148/162 (91.4%)	reference	
Documented bleeding events ^c^	14/171 (8.2%)	0/9 (0.0%)	14/162 (8.6%)	0.00 (0.00–6.01)	

Data are presented as ^1^ n (%). *p*-values were derived from Fisher’s exact test. Effect estimates compare patients with versus without prehospital PE recognition and are reported as odds ratios with exact 95% CI (Fisher’s exact test); for treatment modality and anticoagulation route, each category versus all others. Given the small number of patients with prehospital recognition (n = 9), estimates are imprecise and exploratory. ^a^ Missing in 33 of 171 patients. ^b^ Missing in 43 of 171 patients. ^c^ Documented bleeding events (none meeting ISTH major bleeding criteria); no further differentiation between clinically relevant non-major and minor bleeding was performed. DOAC, direct oral anticoagulant; ISTH, International Society on Thrombosis and Haemostasis.

**Table 6:** Multivariable regression models for prehospital anticoagulation and clinical outcomes.

	ICU Admission n = 168			In-Hospital Mortality n = 171			ICU Length of Stay ^a^ n = 85		
Characteristic	OR (95% CI)	95% CI	*p*-Value	OR (95% CI)	95% CI	*p*-Value	% Change	95% CI	*p*-Value
Prehospital anticoagulation	3.66	1.39, 10.91	0.008	1.51	0.44, 5.23	0.46	13.2	−54.4, 180.9	0.79
Age, per year	0.99	0.97, 1.01	0.11	1.04	1.00, 1.08	0.061	−3.4	−5.9, −0.9	0.01
NEWS2 score, per point	1.26	1.12, 1.41	<0.001	1.36	1.2, 1.56	<0.001	1.6	−8.2, 12.4	0.76

ICU admission and in-hospital mortality were analyzed in all patients with complete data on the variables of the respective model (ICU admission, n = 168, 3 excluded owing to missing ICU admission status; in-hospital mortality, n = 171, 0 excluded). ICU duration was analyzed in all 85 patients admitted to the ICU at any time during hospitalization. Events: ICU admission, 85/168 (50.6%); in-hospital mortality, 25/171 (14.6%). Heparin-treated patients: 28/168 (ICU model), 28/171 (mortality model), 22/85 (duration model). All models were adjusted for age and NEWS2 at EMS arrival. NEWS2 was calculated retrospectively from the records at EMS arrival, all parameters required for its calculation were available. Logistic regression for binary outcomes (OR, 95% CI); sample sizes differ between models owing to missing data (complete-case analysis). ^a^ Duration of ICU treatment in hours. Analyzed via linear regression on log-transformed duration; the percentage change, 100 × [exp(β) − 1], is the percentage change in the duration among patients admitted to the ICU. (percentage change, 95% CI); both adjusted for age and NEWS2 score. CI, confidence interval; ICU, intensive care unit; NEWS2, National Early Warning Score; OR, odds ratio.

## Data Availability

The data presented in this study are available upon request from the corresponding author due to privacy and ethical restrictions.

## Abbreviations

The following abbreviations are used in this manuscript:

CDT	Catheter-directed thrombolysis
COPD	Chronic obstructive pulmonary disease
CPR	Cardiopulmonary resuscitation
DOAC	Direct oral anticoagulant
ED	Emergency department
EMS	Emergency medical services
ESC	European Society of Cardiology
ICD	International Classification of Diseases
ICU	Intensive care unit
IMC	Intermediate care unit
ISTH	International Society on Thrombosis and Haemostasis
mmHg	Millimeters of mercury
NEWS2	National Early Warning Score
PE	Pulmonary embolism
PERC	Pulmonary Embolism Rule-out Criteria
PESI	Pulmonary Embolism Severity Index
SPO_2_	Peripheral oxygen saturation
TTE	Transthoracic echocardiography

## Appendix A

**Table A1:** Comorbidities and thrombosis risk factors.

Characteristic	N = 171 ^1^
Prior venous thrombosis (>3 months)	10 (5.8%)
Active malignancy, n (%)	12 (7.0%)
Prior malignancy	8 (4.7%)
Immobilization	2 (1.2%)
Surgery within past year	2 (1.2%)
Heart failure	15 (8.8%)
Atrial fibrillation	9 (5.3%)
Coronary artery disease	25 (14.6%)
Atherosclerosis	1 (0.6%)
Chronic kidney disease	8 (4.7%)
Hypothyroidism	11 (6.4%)
COPD	21 (12.3%)
Pulmonary fibrosis	2 (1.2%)
Asthma	8 (4.7%)
Stroke	8 (4.7%)
Epilepsy	3 (1.8%)
Infectious disease	2 (1.2%)
Psychiatric disease	1 (0.6%)
Gastrointestinal disease	9 (5.3%)
Other neurological disease	12 (7.0%)
No known comorbidities	28 (16.4%)

^1^ n (%).

**Table A2:** Prior medication at hospital presentation.

Characteristic	N = 171 ^1^
Anticoagulation	
DOAC	4 (2.3%)
Antiplatelet therapy	
Aspirin	39 (22.8%)
Other antiplatelet	2 (1.2%)
Other medication	
Heart failure medication	89 (52.0%)
Antiarrhythmic medication	1 (0.6%)
Lipid-lowering medication	38 (22.2%)
Other cardiovascular medication	26 (15.2%)
Antidiabetic medication	13 (7.6%)
Thyroid medication	21 (12.3%)
Other medication	36 (21.1%)

^1^ n (%).

**Table A3:** Assessment of the proportional hazards assumption and sensitivity analyses for prehospital predictors of time to CTPA acquisition.

		Cox Regression, HR (95% CI)				AFT, TR (95% CI)
Variable	Schoenfeld Test, *p*	Model 1 Primary	Model 2 Stratified by Hypoxemia	Model 3 Stratified by Physician	Model 4 Stratified by Both	Model 5 AFT Log-Normal
Age, per year	0.831	1.00 (0.99–1.01) *p* = 0.556	1.01 (1.00–1.02) *p* = 0.229	1.00 (0.99–1.02) *p* = 0.486	1.01 (1.00–1.02) *p* = 0.182	1.00 (0.98–1.01) *p* = 0.563
Male sex	0.496	1.16 (0.81–1.65) *p* = 0.424	1.18 (0.82–1.69) *p* = 0.374	1.11 (0.78–1.59) *p* = 0.560	1.15 (0.80–1.65) *p* = 0.462	0.92 (0.57–1.49) *p* = 0.737
Physician-staffed EMS response	**0.048**	1.37 (0.95–1.99) *p* = 0.096	1.52 (1.04–2.23) *p* = 0.029	—	—	0.57 (0.35–0.94) *p* = 0.028
Hypoxemia (SpO_2_ < 90%)	**<0.001**	0.75 (0.49–1.14) *p* = 0.178	—	0.79 (0.53–1.20) *p* = 0.272	—	1.15 (0.67–1.98) *p* = 0.600
Tachycardia (HR > 100/min)	0.451	0.83 (0.58–1.17) *p* = 0.287	0.81 (0.57–1.16) *p* = 0.246	0.82 (0.58–1.17) *p* = 0.275	0.79 (0.55–1.14) *p* = 0.206	1.39 (0.87–2.22) *p* = 0.171
Oxygen administration	0.107	1.07 (0.71–1.62) *p* = 0.734	1.09 (0.72–1.65) *p* = 0.670	1.06 (0.70–1.60) *p* = 0.787	1.06 (0.70–1.61) *p* = 0.784	1.06 (0.60–1.86) *p* = 0.852
Prehospital anticoagulation	0.145	2.42 (1.40–4.19) *p* = 0.002	2.31 (1.34–3.98) *p* = 0.003	2.28 (1.31–3.96) *p* = 0.004	2.18 (1.24–3.83) *p* = 0.007	0.39 (0.19–0.79) *p* = 0.009
Global PH test	0.004	*p* = 0.004	*p* = 0.237	*p* = 0.021	*p* = 0.307	n/a

n = 144; all patients contributed an event (no censoring). The proportional hazards (PH) assumption was assessed using scaled Schoenfeld residuals; bold type indicates *p* < 0.05 in Model 1. Model 1, primary Cox model; Model 2, Cox model stratified by hypoxemia; Model 3, Cox model stratified by physician-staffed EMS response; Model 4, Cox model stratified by both variables; Model 5, accelerated failure time model with log-normal distribution. Variables used as stratification variables have no estimate (—). A hazard ratio (HR) above 1 indicates a higher instantaneous rate of reaching the diagnostic endpoint; a time ratio (TR) below 1 indicates a shorter time to CTPA acquisition. Of 21 patients classified as anticoagulated, 19 were attended by an emergency physician. All analyses were exploratory. AFT, accelerated failure time; CI, confidence interval; CTPA, computed tomography pulmonary angiography; EMS, emergency medical services; HR, hazard ratio; PH, proportional hazards; SpO_2_, peripheral oxygen saturation; TR, time ratio.
